# Concurrent Presentation of Human Herpesvirus 8-Associated Multicentric Castleman Disease and Kaposi Sarcoma in a Young Patient

**DOI:** 10.7759/cureus.84346

**Published:** 2025-05-18

**Authors:** Sana Tabish, Ann Kurian, Almunther Wael Alhasawi

**Affiliations:** 1 Department of Histopathology, AL-Jahra Hospital, Al Jahra, KWT; 2 Department of Histopathology, Al-Jahra Hospital, Al Jahra, KWT; 3 Department of Public Health, Al-Jahra Hospital, Al Jahra, KWT

**Keywords:** human immunodeficiency virus, kaposi sarcoma, kaposi sarcoma herpesvirus, lymphadenopathy, multicentric castleman disease, plasmablast

## Abstract

Castleman disease and Kaposi sarcoma are uncommon conditions that can occur in association with human immunodeficiency virus (HIV) infection. We report a case of a 26-year-old man newly diagnosed with acquired immune deficiency syndrome who presented with fever, nocturnal chills, generalized lymphadenopathy, hepatosplenomegaly, anemia, and thrombocytopenia. Imaging revealed widespread lymphadenopathy and bone marrow hypermetabolism. Histopathological examination of an excised cervical lymph node demonstrated atrophic follicles with involuted germinal centers and an expanded mantle zone containing scattered plasmablasts - features consistent with Kaposi sarcoma herpesvirus/human herpesvirus 8 (KSHV/HHV-8)-associated multicentric Castleman disease, with microscopic foci of Kaposi sarcoma featuring spindle cell proliferation with slit-like vascular channels. Immunohistochemical staining confirmed HHV-8 positivity in plasmablasts and spindle cells of Kaposi sarcoma. This case highlights the importance of a thorough evaluation of HIV-positive patients presenting with lymphadenopathy to ensure early recognition of rare but clinically significant conditions requiring aggressive management. Recognition of concurrent HHV-8-associated Castleman disease and Kaposi sarcoma is critical, as treatment strategies differ and outcomes may be influenced by early therapeutic intervention.

## Introduction

Castleman disease (CD) was first described in 1954 by Benjamin Castleman, according to Mremi et al. [1]. It is a heterogeneous group of disorders, clinically divided into unicentric Castleman disease (UCD) and multicentric Castleman disease (MCD). Morphologically, CD is classified into the following three variants: hyaline vascular, plasma cell, and mixed [2]. UCD involves a single lymph node or a tightly packed group of lymph nodes, while MCD typically presents with multifocal lymphadenopathy and systemic symptoms. Subtyping of MCD depends on the identifiable cause, and human herpesvirus 8 (HHV-8) is a major etiologic agent in both immunocompetent and immunocompromised patients. In immunocompromised patients, MCD is particularly associated with human immunodeficiency virus (HIV)/acquired immune deficiency syndrome (AIDS) [2,3]. MCD has a poor prognosis and is associated with a rapidly fatal clinical course [3].

Kaposi sarcoma (KS) is a vascular neoplasm of intermediate malignant potential, having the following four clinical subtypes: classic (Mediterranean with high prevalence of HHV-8), endemic (African), iatrogenic (transplant-related), and epidemic (HIV/AIDS-related) [4]. KS typically presents with cutaneous lesions; however, lymphadenopathic or extracutaneous involvement has been observed in patients with HIV infection [5]. We present a case of a young man newly diagnosed with HIV infection who was found to have concurrent KS and Kaposi sarcoma herpesvirus/human herpesvirus 8 (KSHV/HHV-8)-associated multicentric Castleman disease.

## Case presentation

A 26-year-old man was recently diagnosed with AIDS caused by HIV infection. He presented with a history of fever and nocturnal chills persisting for more than six weeks. Initial laboratory investigations revealed progressively worsening thrombocytopenia and anemia, with a negative septic workup. His absolute lymphocyte count was 1614 cells/μL. The CD4 count at the time of diagnosis was 201 cells/μL (12%), with a CD4/CD8 ratio of 0.17%, and an HIV viral load of 462,190 copies/mL.

**Figure 1:**
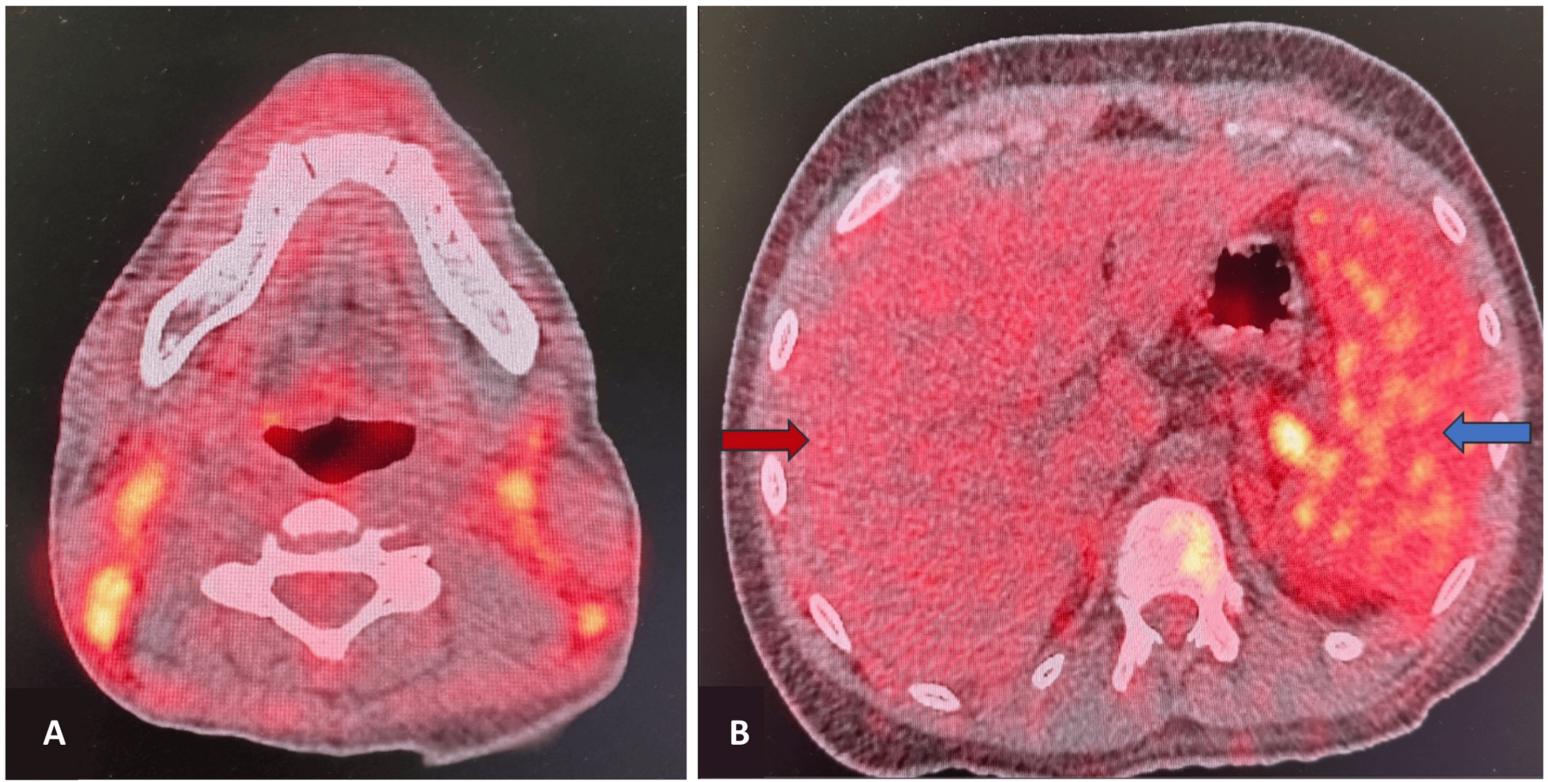
PET-CT findings in cervical lymph nodes and spleen. (A) PET-CT scan showing cervical lymphadenopathy with increased fluorodeoxyglucose uptake and (B) increased fluorodeoxyglucose uptake in the spleen (blue arrow) compared to the liver (red arrow). PET-CT: positron emission tomography-computed tomography

The patient underwent positron emission tomography-computed tomography using fluorodeoxyglucose (FDG), which revealed cervical, axillary, mediastinal, abdominal, and pelvic lymphadenopathy. Multiple hypermetabolic bilateral small cervical lymph nodes were identified, involving levels I-V, with a maximum standardized uptake value (SUVmax) of 6.2 (Figure 1, panel A). Hepatosplenomegaly was evident with increased FDG uptake in the spleen (SUVmax 6.02) compared to the liver (SUVmax 3.3) (Figure 1, panel B). The axial and proximal appendicular skeleton also demonstrated hypermetabolic activity in the bone marrow. The possibility of lymphoma was considered based on lymphadenopathy, hepatosplenomegaly, and increased FDG uptake in the bone marrow. An excisional cervical lymph node biopsy was performed, and the specimen was sent for histopathological examination to rule out a lymphoproliferative disorder.

**Figure 2:**
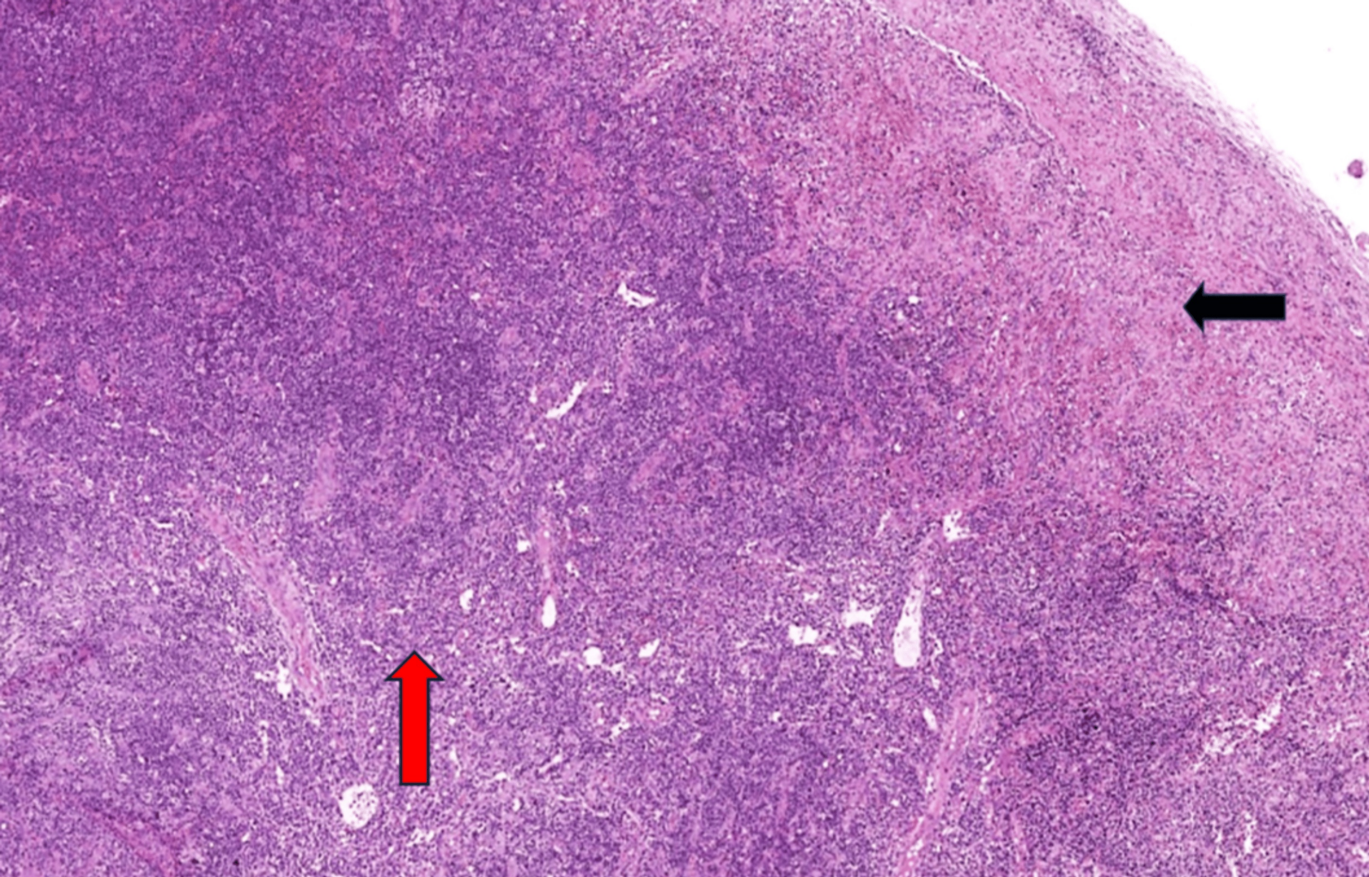
Histopathological findings in cervical lymph node. Effaced lymph node with scattered hyalinized vessels, expansion of the interfollicular area by plasma cells (red arrow), and subcapsular spindle cell proliferation (black arrow) (hematoxylin and eosin stain, 4× magnification).

**Figure 3:**
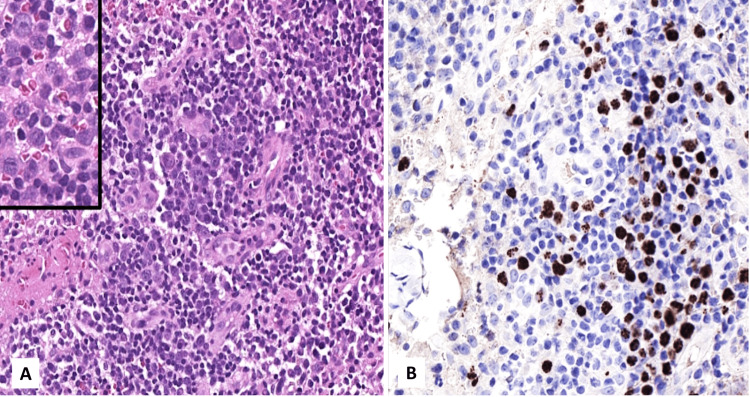
Cervical lymph node showing features of HHV-8 associated Castleman disease. (A) Large atypical plasmablasts (hematoxylin and eosin stain, 10× magnification); inset shows atypical plasmablasts at higher magnification (40×). (B) Immunohistochemical staining demonstrating HHV-8 expression in plasmablasts (20× magnification). HHV8: human herpesvirus 8

**Figure 4:**
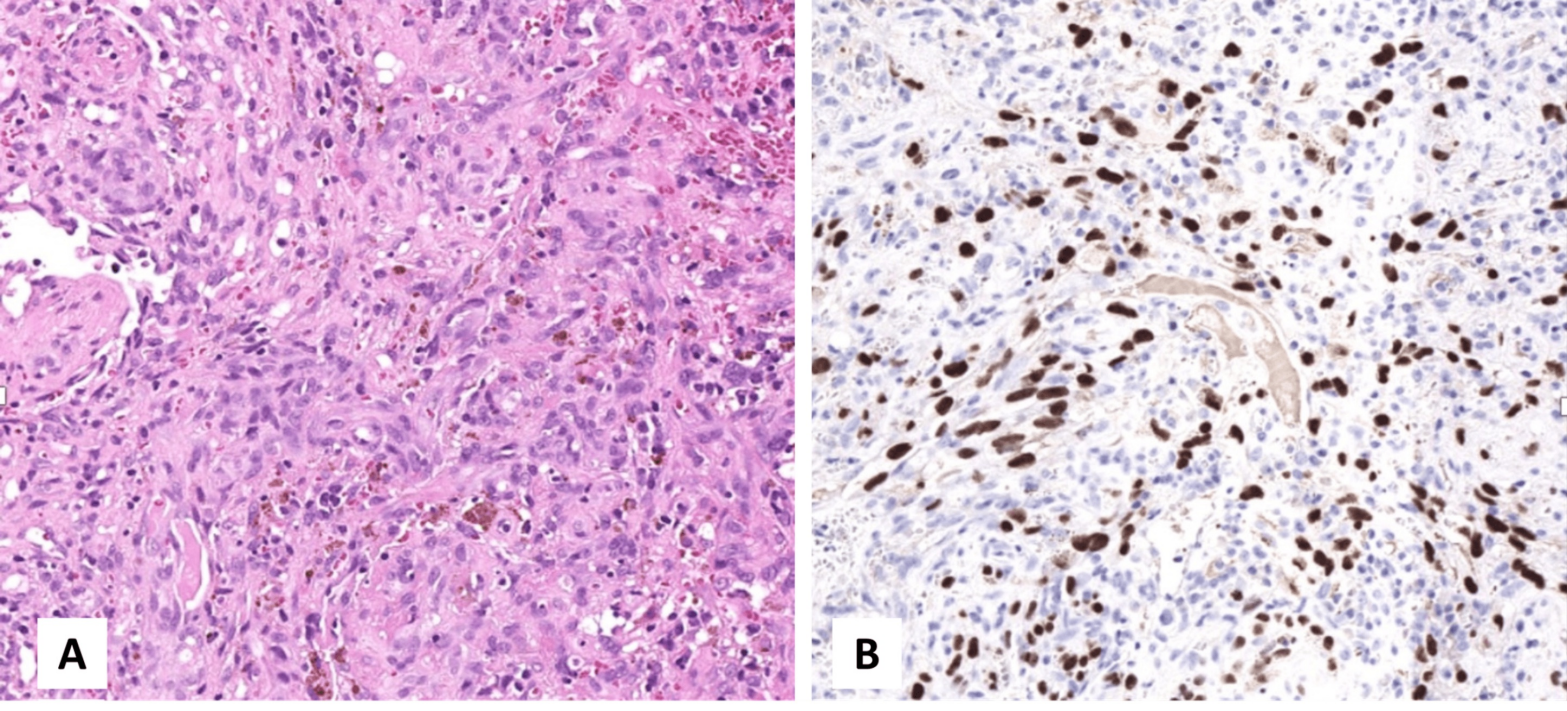
Nodal Kaposi sarcoma. (A) Spindle cell proliferation with slit-like vascular channels (hematoxylin and eosin stain, 10× magnification). (B) Immunohistochemical staining demonstrating HHV-8 expression in spindle endothelial cells (10× magnification). HHV8: human herpesvirus 8

The surgical specimen consisted of an intact lymph node measuring 1.2 × 1.0 × 1.0 cm, with a pale white cut surface. The entire lymph node was submitted for histopathological evaluation. Microscopic examination revealed atrophic follicles with involuted germinal centers and an expanded mantle zone containing scattered plasmablasts (Figure 2 and Figure 3, panel A). Sheets of polyclonal plasma cells expanded the interfollicular area and showed excessive vascular proliferation. The subcapsular area and parenchyma demonstrated foci of spindle cell proliferation with slit-like vascular channels and extravasated red blood cells (Figure 2 and Figure 4, panel A).

Immunohistochemical staining showed that CD20 was positive in residual reactive B cells, and there was no downregulation of the T-cell population on T-cell markers. CD138 was positive in scattered plasma cells, with no evidence of kappa or lambda light chain restriction. HHV-8 was positive in plasmablasts (Figure 3, panel B) and in the endothelial cells of the slit-like vascular proliferations (Figure 4, panel B). Special stains for fungal organisms and *Mycobacterium tuberculosis *were negative. Based on these findings, the case was diagnosed as KSHV/HHV-8-associated multicentric Castleman disease with microscopic foci of KS. After diagnosis, the patient was referred to the oncology center for further management plan (HHV-8-related therapy). However, the patient died before the initiation of treatment.

## Discussion

KSHV, also known as HHV-8, was first identified in 1994 in a patient with AIDS and KS [6]. Subsequent studies found HHV-8 to be associated with other B-cell proliferations, including primary effusion lymphoma, most cases of MCD, HHV-8-positive diffuse large B-cell lymphoma not otherwise specified (NOS), and HHV-8-positive germinotropic lymphoproliferative disorder [7]. KSHV/HHV-8-associated disorders represent a heterogeneous group of proliferations with a wide pathological spectrum, ranging from reactive hyperplasia to aggressive lymphoma [6].

Subtyping of MCD is based on etiological factors and includes HHV-8-positive MCD, HHV-8-negative MCD, and idiopathic MCD. HHV-8 is the major etiological agent, particularly in immunocompromised patients, most notably those with HIV/AIDS [2,8]. In contrast, MCD in immunocompetent individuals is typically HIV-negative and occurs more frequently in HHV-8 endemic areas, such as sub-Saharan Africa and the Mediterranean region [8]. Cases without an identifiable cause are categorized as idiopathic MCD [9]. The pathogenesis of HHV-8-positive MCD involves viral infection of B-cells within the mantle zone. These infected cells produce viral interleukin-6 (IL-6) and activate the human IL-6-induced cellular pathway, contributing to the clinical and pathological features of the disease [1,3,9].

The mean age of onset for MCD is typically in the fifth to sixth decade of life, although in patients with HIV infection, it often presents at a younger age, as observed in this case. MCD usually presents with multifocal lymphadenopathy and systemic symptoms [2,9]. These systemic symptoms result from a systemic inflammatory response, including organomegaly, cytopenias, multiorgan dysfunction, constitutional symptoms, and elevated acute-phase proteins [9]. Patients may also experience symptoms related to complications or comorbidities, such as lymphoma or KS. In this case, the patient presented with fever, nocturnal chills, generalized lymphadenopathy, hepatosplenomegaly, and progressively worsening thrombocytopenia and anemia.

Morphologically, Castleman disease overlaps with other conditions such as HIV-associated lymphadenopathy, autoimmune diseases, Hodgkin lymphoma, and plasmacytoma [10]. In the present case, based on morphology and HHV-8 immunopositivity, differential diagnoses included HIV-associated lymphadenopathy and MCD. However, the presence of atretic follicles, an expanded mantle zone with scattered plasmablasts, prominent vascular proliferation, and sheets of plasma cells in the interfollicular areas supported the diagnosis of MCD, plasma cell variant.

Patients with HHV-8-associated MCD have an increased risk of developing other HHV-8-related disorders, including KS, primary effusion lymphoma, and HHV-8-positive diffuse large B-cell lymphoma, NOS. In our case, additional pathological findings included subcapsular and parenchymal foci of KS, characterized by spindle cell proliferation, slit-like vascular channels, and extravasated red blood cells. These spindle cells demonstrated immunoreactivity for HHV-8. Considering the spindle cell morphology and the patient’s history of HIV infection, mycobacterial spindle cell pseudotumor was also in the differential diagnosis. However, the absence of a storiform pattern, negative acid-fast bacilli on Ziehl-Neelsen stain, and positive HHV-8 staining in spindle cells ruled out this possibility.

According to Vega et al., concurrent presentation of HHV-8-associated Castleman disease with KS has been occasionally observed [6]. Limited literature exists regarding the concurrent presence of KS and HHV-8-associated MCD. A study by Gonzalez-Farre et al. investigated a cohort of 66 patients with HHV-8 infection and found that 40% had nodal KS along with MCD [7]. Although MCD in patients with HIV infection is typically thought to arise after a prolonged period of antiretroviral therapy, usually around three years, most patients in the study by Gonzalez-Farre et al. developed MCD shortly after their HIV diagnosis [7]. Similarly, our patient presented with concurrent KS and HHV-8-associated Castleman disease at the time of HIV diagnosis.

MCD is an aggressive inflammatory disorder with a poor prognosis. Antiretroviral therapy (ART) used for the treatment of all HIV patients is insufficient in patients with HHV-8-associated Castleman disease. To achieve long-term remission, ART must be used in conjunction with targeted therapy against HHV-8-associated CD. Treatment for HHV-8-associated Castleman disease typically involves rituximab; however, in patients with concurrent KS, rituximab leads to exacerbation of KS. In these cases, the addition of doxorubicin, an FDA-approved medication for KS, helps control the exacerbation and may even lead to complete regression of KS. Doxorubicin not only helps in the treatment of KS but also targets plasmablasts in HHV8-associated Castleman disease [10,11].

## Conclusions

This study describes a newly diagnosed HIV patient with rare concurrent KS and KSHV/HHV-8-associated multicentric Castleman disease. This case highlights the importance of thoroughly investigating HIV-positive patients due to their increased risk of developing a broad range of inflammatory, non-neoplastic, and neoplastic conditions. Early and accurate recognition of both HHV-8-associated Castleman disease and KS is critical, as treatment strategies differ. Patients with concurrent disease require more aggressive therapy, typically combining rituximab and doxorubicin. Furthermore, HHV-8-associated Castleman disease carries a heightened risk of progression to aggressive lymphoma, emphasizing the need for prompt diagnosis and management.
